# Case report: Lymph node metastasis of pelvic alveolar rhabdomyosarcoma diagnosed by fine needle aspiration cytology

**DOI:** 10.3389/fonc.2024.1340865

**Published:** 2024-05-17

**Authors:** Xinyu Hu, Chenxin Huang, Qiyuan Li, Baolin Wu, Chuyun Yue, Xueying Su

**Affiliations:** ^1^ Department of Pathology, West China Hospital, Sichuan University, Chengdu, China; ^2^ Department of Radiology and MR Research Center, Functional and Molecular Imaging Key Laboratory of Sichuan Province, West China Hospital, Sichuan University, Chengdu, China; ^3^ Medical Imaging Science, The Second Clinical Medical School of ChongQing Medical University, ChongQing, China

**Keywords:** fine needle aspiration cytology, alveolar rhabdomyosarcoma, lymph node metastasis, FNA (fine needle aspiration), ARMS

## Abstract

Rhabdomyosarcoma (RMS) is a common soft tissue malignant tumor, especially in young patients. Alveolar rhabdomyosarcoma (ARMS) is a subtype of RMS that is prevalent in adolescents. This malignant tumor usually develops in the extremities and can also involve the trunk, perineum, and pelvis. Now, we report a rare case of pelvic lymph node metastatic alveolar RMS in a young patient, which was determined by fine needle aspiration cytology (FNAC). To the best of our knowledge, this is the first case in which the definite diagnosis of ARMS was initially made by FNAC.

## 1 Introduction

RMS is the most common soft tissue sarcoma in children, accounting for about half of all soft tissue tumors, which are usually localized in the extremities, trunk, head, and neck (1, 2). It is rarely observed in adults and only accounts for 3% of adult soft tissue sarcomas, which has a significantly worse prognosis compared to children (3). RMS can be divided into two prevalent subtypes: embryonal rhabdomyosarcoma (ERMS) and ARMS. In addition, rare subtypes include pleomorphic rhabdomyosarcoma (PRMS) and spindle cell/sclerosing rhabdomyosarcoma (SRMS) (4). ARMS occurs most frequently in the deeper parts of the extremities and can also involve the trunk, perineum, and pelvis. In terms of age, ARMS accounts for approximately 20% of all pediatric RMS and is most common in adolescents and young adults between the ages of 10 and 25 (5).

FNAC is a minimally invasive way of obtaining diseased tissue and has achieved widespread application in the diagnosis of many sites and types of tumors (6–8). Combined with various assistive techniques, the correctness of the FNAC diagnosis is comparable to that of histopathological biopsy for some kinds of tumors (6, 9). FNAC of suspicious lymph nodes also provides additional information on tumor staging, which can better guide subsequent treatment (10, 11).

## 2 Case presentation

### 2.1 Case history and image logical findings

A 20-year-old male patient was admitted to the hospital with the chief complaint of “recurrent hematuria for 1 month, aggravated by fever and abdominal pain for 10 days”. A physical examination revealed that the patient had lower abdominal tenderness, bilateral scrotal swelling, and slight perianal swelling with squeezing pain. A digital rectal examination revealed that the patient’s prostate was enlarged with a soft mass nearby, had a fluctuating sensation, and was poorly defined. A small amount of discharge was present on the finger inspection.

A male urologic ultrasound was performed on the patient, which revealed bilateral hydronephrosis with uneven enlargement of the prostate, suggesting infectious disease. An enhanced CT scan of the lower abdomen and pelvis was performed subsequently, which suggested lymphoma or an infectious disease.

**Figure 1:**
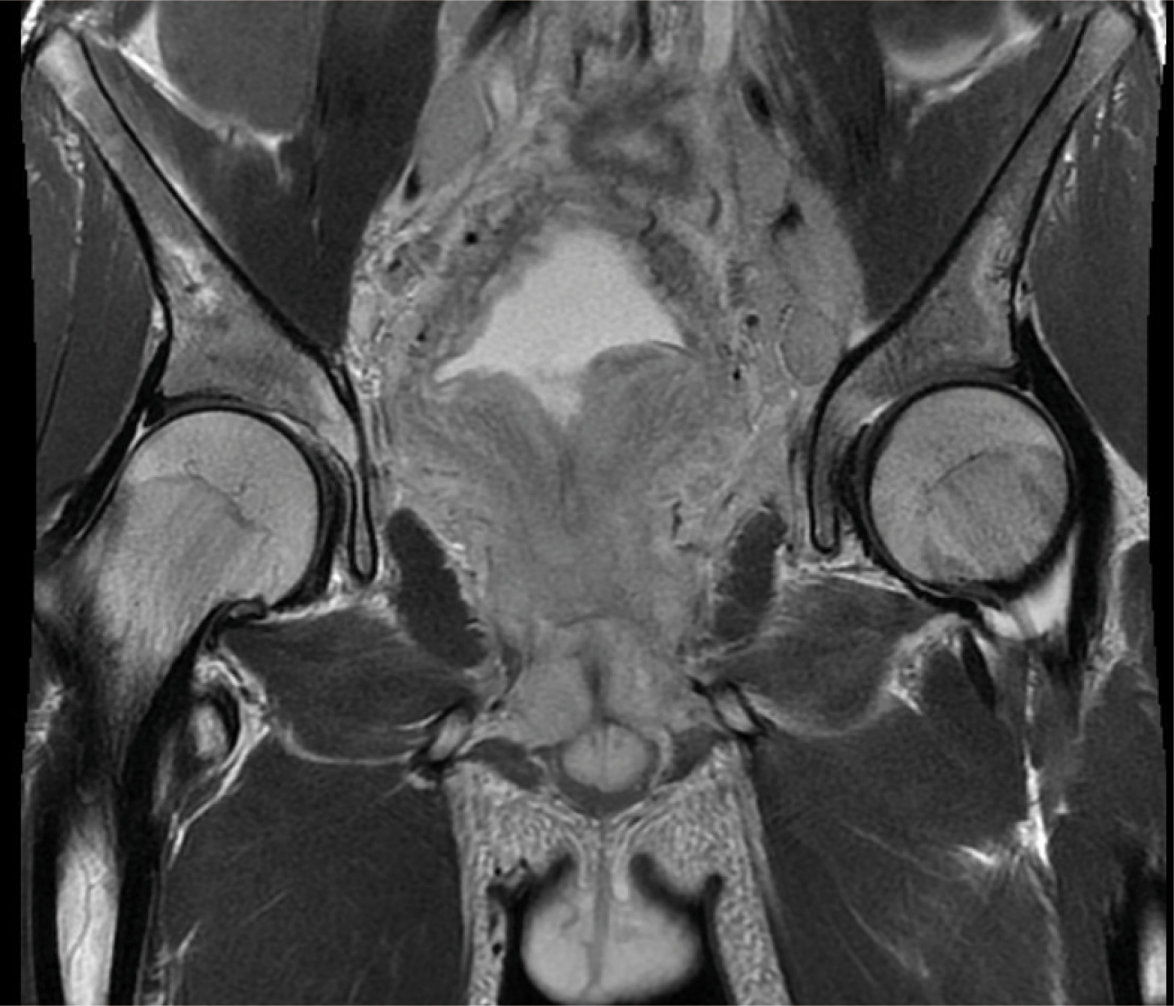
The MRI figure showed diffuse abnormal signals in the pelvis and pelvic extraperitoneal space. The prostate was enlarged, and there was extensive swelling of the bladder, rectum, sigmoid colon bowel wall, pelvic floor muscle groups, pelvic peritoneum, and pelvic sacral fascia. Lymph nodes in the pelvic and bilateral inguinal regions were increased and partly enlarged.

To further investigate the patient’s pelvic lesions, an MRI enhancement scan was carried out, which showed diffuse abnormal signals in the pelvis and pelvic extraperitoneal space. The prostate was enlarged with extensive swelling of the bladder, rectum, sigmoid colon bowel wall, pelvic floor muscle groups, pelvic peritoneum, and pelvic sacral fascia. Lymph nodes in the pelvic and bilateral inguinal regions were increased and enlarged. Lymphoma, or infection, was considered by the MRI (**Figure 1**).

### 2.2 Ultrasound-guided FNAC

Fine needle aspiration (FNA) was performed for this patient with ultrasound guidance. Ultrasound showed an enlarged lymph node measuring about 33 mm × 16 mm × 16 mm, adjacent to the left iliac vessel, which had unclear corticomedullary demarcation and a rich internal blood flow signal. After local anesthesia, multipoint aspiration of a left iliac perivascular lymph node was conducted by using a 23-G needle under ultrasonic monitoring. Two smears were made and fixed with 95% alcohol immediately. The residue material in the needle was collected and fixed in 10% neutral buffer formalin to make a cell block. The smears were subjected to Pap staining; 4 μm sections were taken from the formalin-fixed and paraffin-embedded cell block and then stained for hemoglobin and eosin (H&E).

### 2.3 Immunocytochemistry and *in situ* hybridization

Immunocytochemistry (ICC) was conducted on 4 μm sections taken from the cell block using a Roche VENTANA ULTRA automated immunostaining machine following the steps of EnVision. The following antibodies were employed: Desmin, Myogenin, MyoD1, CD56, Syna, ALK-1, LCA, PCK, CD99, MPO, S100, HMB45, CD79a, CD20, CD3, CD30, TIA-1, CD117, Granzyme B, OCT3/4, CgA, and Ki-67. *In situ* hybridization (ISH) test was performed for EBER1/2 also using a 4μm section of the cell block.

### 2.4 Fluorescence *in situ* hybridization

A FOXO1 (FKHR) two-color break-apart probe (Abbott Molecular, Abbott Park, IL, USA) was used, which consisted of a 720-kb probe labeled with the Spectrumgreen™ fluorophore located proximal to the FOXO1 gene as well as a 650-kb probe labeled with the SpectrumOrange™ fluorophore located at 13q14 distal to the FOXO1 gene.

## 3 Results

**Figure 2:**
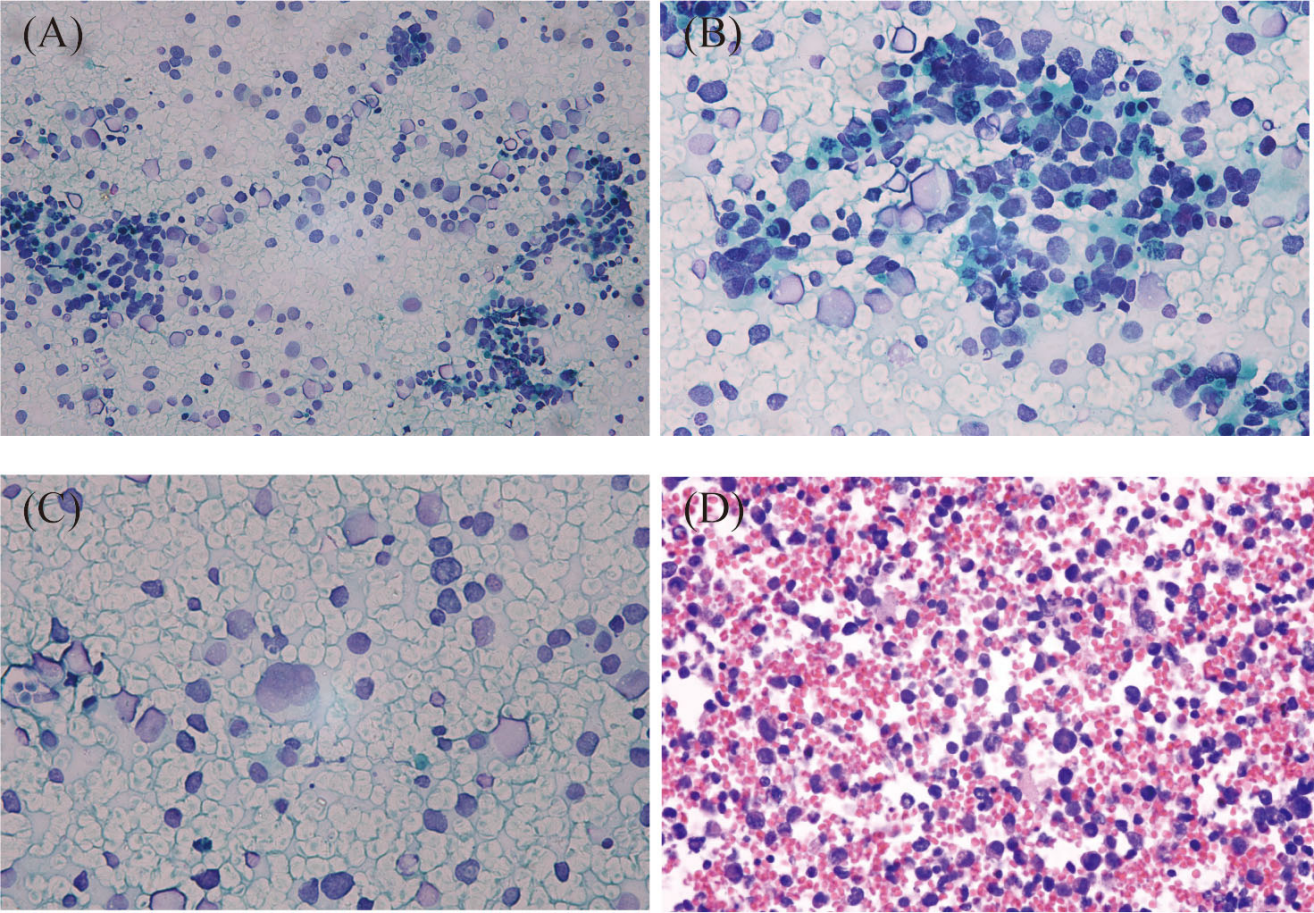
Cytologic features of metastatic alveolar rhabdomyosarcoma of the lymph nodes. Most tumor cells were scatter-distributed, and some loosely packed clusters could also be found in the background of lymphocytes **(A)**, Papanicolaou staining, ×200). The nuclei were irregular and eccentric, with coarse chromatin and small nucleoli **(B)**, Papanicolaou staining, ×400). Some binucleated and multinucleated cells were present **(C)**, Papanicolaou staining, ×400). Scattered small blue-round cells with rhabdoid features were seen on the cell block section. The nuclei were deeply stained, and tumor giant cells were observed in this figure **(D)**, HE staining, ×400).

The smears and cell block sections were highly cellular. Many small blue-round cells with high N/C ratios were present, with some small lymphocytes in the background. Most abnormal cells were scatter-distributed, and some loosely packed clusters could also be found. The nuclei were irregular and eccentric, with coarse chromatin and small nucleoli, which showed marked heteromorphism. Apoptosis and mitosis were frequent. Some binucleate and multinucleate abnormal cells were observed (**Figure 2**).

**Figure 3:**
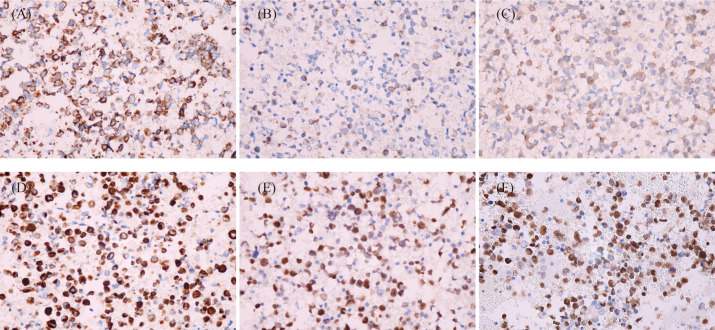
Immunocytochemistry of cell block sections (EnVision, ×400). **(A)** Tumor cells showed CD56-positive stains. **(B)** Tumor cells showed Syna-positive stains. **(C)** Tumor cells showed ALK-positive stains. **(D)** Tumor cells showed Des-positive stains. **(E)** Tumor cells showed Myogenin-positive stains. **(F)** Tumor cells showed MyoD1-positive stains.

**Figure 4:**
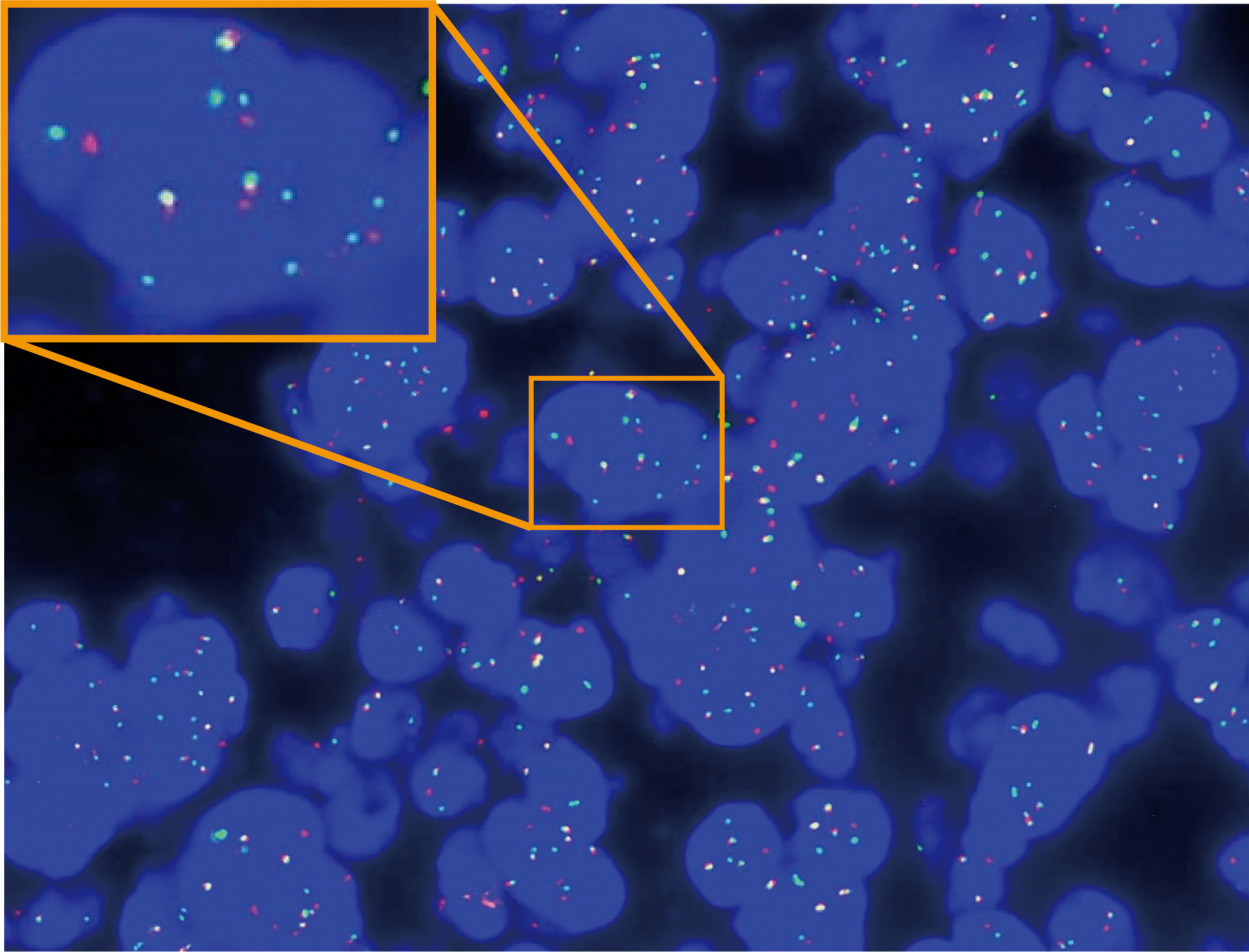
FISH-detected FOXO1 (FKHR) gene translocation.

Based on the cytological morphology and imageological findings, lymphohematopoietic tissue tumors were first considered, and relevant immunophenotyping tests, *in situ* hybridization tests, and gene rearrangement tests were performed. However, ICC showed LCA, CD3, CD20, CD79a, CD30, TIA-1, Granzyme B, and MPO were all negative, while only CD56, ALK, and Ki-67 (80%) were positive. ISH test showed EBER1/2 (−). Gene rearrangement tests for IgH and TCRG were all negative. According to the above test results, lymphohematopoietic tissue tumors were basically ruled out, and then metastatic tumors of the lymph node were considered. The second round immunophenotyping test showed PCK, CD99, OCT3/4, CD117, S100, and HMB45 were all negative while only Syna was positive, which can rule out metastatic carcinoma, germ cell tumor, melanoma, and primitive neuroectodermal tumor. Combined with the patient’s age, cytological morphology, and the results of the first two rounds of immunophenotyping tests (CD56+, Syna+, and ALK+), metastatic RMS was considered, and the third round of immunophenotyping test was carried out, which showed Desmin, Myogenin, and MyoD1 were all positive (**Figure 3**). So, the diagnosis of metastatic RMS was made. To further determine the subtype of RMS, the Fluorescence *in situ* hybridization (FISH) test for FOXO1 (FKHR) gene rearrangement was performed, and the FOXO1 gene translocation was detected (**Figure 4**). At last, metastatic ARMS of the left paravascular iliac lymph node was diagnosed by FNAC, and the pelvic lesion was considered ARMS by the clinicians.

However, the surgical treatment and core needle aspiration biopsy were not put into practice because of the wide extent of the pelvic lesion and the poor general condition of this patient. The patient chose to receive chemotherapy and symptomatic supportive treatment. After two courses of chemotherapy, the patient was discharged. Follow-up was conducted 6 months later, and we learned the patient turned worse and only received symptomatic supportive treatment such as pain relief and nutrition at home. After 3 months, the patient was lost to follow-up.

## 4 Discussion

RMS is one of the most common sarcomas with lymph node metastasis, while other types of sarcomas usually spread via blood vessels (12, 13). It can be categorized into four subtypes: embryonic, alveolar, polymorphic, and sclerosing RMS. In terms of cytological morphology, most RMS share certain features, such as high cellularity with predominantly small, round, single blue cells with occasional loosely packed clusters (14, 15).

ERMS consists of stellate, small blue-round or short spindle-shaped mesenchymal cells that are morphologically diverse and show a mixed cellular pattern. Tumor cells in ARMS are larger and have more cytoplasm than those from the ERMS; binucleate or multinucleate cells are more commonly seen; and alveolar structures may be present. Tumor cells in PRMS are highly variable in size and morphology, exhibiting marked pleomorphism and larger rhabdomyoblastic cells. In SRMS, a dense sclerotic background is typically seen. However, a reliable subclassification of RMS cannot be made only by FNA smears due to the lack of histological structure. Moreover, the ARMS also needs to be differentiated from other small blue-round cell tumors, including lymphoma, Ewing/PNET, neuroblastoma, and desmoplastic small round tumors (5). The rhabdoid feature can provide a hint and help with the differential diagnosis, which is more obvious on the cell block sections. However, it is not easy to be observed on Pap staining and may lead to misdiagnosis. So, a cell block should be made, and an ICC test should be used for the differential diagnosis.

Immunohistochemical detection of skeletal muscle phenotype-specific markers, including Desmin, Myogenin, and MyoD1, can accurately lead to a diagnosis of RMS. However, the expression of Desmin, Myogenin, and MyoD1 differs across the subtypes. It has been shown that ERMS showed patchy staining of Myogenin, whereas ARMS showed diffuse staining, which may help differentiate ERMS and ARMS (16). As the two rare subtypes of RMS, PRMS only focally expresses Myogenin and MyoD1 (17), SRMS often diffusely expresses MyoD1, and focally expresses Myogenin, while both of them predominantly express Desmin (18). It is worth noting that some cases of ARMS can also express Syn, CD56, AE1/AE3, and ALK, which makes it necessary to distinguish ARMS from small cell carcinomas and neuroendocrine carcinomas (19, 20). The tumor cells in this case diffusely expressed Desmin, Myogenin, and MyoD1, which can lead to the diagnosis of ARMS. Furthermore, the positive stains of Syna, CD56, and ALK were also important clues to the diagnosis of ARMS.

The genetic characteristics can also help identify the subtypes of RMS (21). It has already been discovered that tumor cells of ARMS usually contain *t*(2;13) or *t*(1;13) translocations producing PAX3-FOXO1 or PAX7-FOXO1 fusion genes, which are absent in other subtypes of RMS. In this case, though the tests for these two fusion genes were not performed, the break-apart of the FOXO1 gene at locus 13q14 was detected by FISH, which further strengthens the possibility of an ARMS diagnosis.

FNAC is used to examine neoplastic and non-neoplastic lesions at different sites. Because of the tiny caliber of the puncture needle, fine needle aspiration minimizes the risks of blooding and tumor dissemination. The real-time guidance of the ultrasound allows the diseased tissue to be accessed more accurately. Many soft tissue tumors have distinctive clinical characteristics, such as specific age ranges and predilection body sites, which can be helpful in making a diagnosis. However, it still faces great challenges when only cytological materials are available and a definite diagnosis must be made (22). With the advancement of modern pathology, some cases that are difficult to diagnose morphologically can be determined in combination with immunophenotyping and genetic features. It is not uncommon in clinical practice to make a diagnosis of RMS by FNAC (23, 24), but only a few studies have discussed the cytologic diagnosis of metastatic ARMS in lymph nodes. Those three cases had been reported arising from the nasal cavity, paranasal sinus, or nasal vestibule with metastasis to cervical lymph nodes. However, none of these cases was proved as ARMS just by using cytological materials because histological biopsies were available (25–27).

In this case, the surgical excisional biopsy and core needle aspiration biopsy could not be performed due to the poor general condition of the patient. When a histological biopsy is unavailable, FNA is the only method to get the diseased tissue, and the cytological diagnosis is very essential in instructing the choice of subsequent treatment and prognostic judgment. In such a situation, the cytological diagnosis should be made with great caution. Communication between the cytopathologist, radiologist, and clinician is important to ensure an optimal interpretation and avoid diagnostic pitfalls. A correct diagnosis should be made based on the clinical history, imaging features, cytomorphology, immunophenotypes, and molecular detection. Even so, many soft tissue tumors still cannot be diagnosed by FNAC without histological confirmation. However, this case had typical cytological features, including small, blue-round tumor cells, rhabdomyoblasts, and binucleate or multinucleate abnormal cells. Immunophenotyping tests showed positivity of tumor cells for Desmin, Myogenin, MyoD1, Syna, CD56, and ALK. Moreover, the break-apart of the FOXO1 gene at locus 13q14 was detected by FISH. So, a definite diagnosis of ARMS can be made by FNAC.

## 5 Conclusions

In conclusion, though sarcomas generally develop bloodstream metastases, RMS is relatively prone to lymph node metastasis. If small blue-round cells are found when performing FNAC on suspicious lymph nodes of children or young adults, lymph node metastasis of RMS should be considered in addition to other malignant tumors in this age group. When histological biopsy is unavailable, FNAC can play an essential role in making the diagnosis. The clinical history, imaging features, cytomorphology, immunophenotyping, and molecular testing should be combined together to make a correct diagnosis, which can guide the following treatment and prognostic judgment.

## Data availability statement

The raw data supporting the conclusions of this article will be made available by the authors, without undue reservation.

## Ethics statement

The studies involving humans were approved by West China Hospital of Sichuan University Biomedical Research Ethics Committee. The studies were conducted in accordance with the local legislation and institutional requirements. Written informed consent for participation was not required from the participants or the participants’ legal guardians/next of kin in accordance with the national legislation and institutional requirements. Written informed consent was obtained from the individual(s) for the publication of any potentially identifiable images or data included in this article.

## Author contributions

XH: Writing – original draft, Writing – review & editing. CH: Writing – original draft, Writing – review & editing. QL: Writing – original draft. BW: Writing – original draft. CY: Writing – original draft. XS: Writing – original draft, Writing – review & editing.
